# Androgen receptor antagonist flutamide modulates estrogen receptor alpha expression in distinct regions of the hypospadiac rat penis

**DOI:** 10.3389/fendo.2025.1654965

**Published:** 2025-09-12

**Authors:** Emilie Elmelund, Monica K. Draskau, Marie Berg, Ida W. Strand, Jay R. Black, Marta Axelstad, Andrew J. Pask, Terje Svingen

**Affiliations:** ^1^ National Food Institute, Technical University of Denmark, Kongens Lyngby, Denmark; ^2^ School of Geography, Earth and Atmospheric Sciences, The University of Melbourne, Melbourne, VIC, Australia; ^3^ Trace Analysis for Chemical, Earth and Environmental Sciences (TrACEES) Platform, The University of Melbourne, Melbourne, VIC, Australia; ^4^ School of BioSciences, The University of Melbourne, Melbourne, VIC, Australia

**Keywords:** androgen receptor, endocrine disrupting chemicals, estrogen receptor, genital tubercle, hypospadias, penis development

## Abstract

**Introduction:**

Intrauterine exposure to endocrine disrupting chemicals (EDCs), particularly anti-androgens, has been implicated in hypospadias by disrupting fetal masculinization of the genital tubercle (GT). Other pathways, including estrogen signaling, may also contribute but remain poorly characterized, especially in rats – a key model in chemical toxicity testing. Estrogen signaling has also been linked to hypospadias in mice, raising questions about androgen-estrogen interactions in guiding GT differentiation.

**Methods:**

We induced hypospadias in male rat offspring via intrauterine exposure to the antiandrogenic drug flutamide and characterized androgen and estrogen receptor expression.

**Results:**

We observed key structural and transcriptional changes in the developing penis, including altered estrogen receptor a (ERa, Esr1) expression. Notably, beyond this established androgen-estrogen relationship in hormone-sensitive tissues, anti-androgenic exposure also induced spatial changes in Esr1 expression in specific regions of the GT.

**Discussion:**

Future toxicological testing using new approach methodologies (NAMs) should consider androgen-estrogen balance and crosstalk in reproductive tissues as a mechanism of action.

## 1 Introduction

Hypospadias is a genital malformation in males where the urethra opens on the ventral side of the penis instead of at the tip. It is one of the most common congenital abnormalities in newborn males, with an incidence rate up to ~1/125 in some Western countries and with increasing prevalence over the last 30 years (1–3). Some cases of hypospadias have a known genetic basis, but in most cases, the underlying cause is unknown. The rising prevalence and unknown etiology suggest environmental factors may be causally involved (4). Indeed, developmental exposure to endocrine disrupting chemicals (EDCs) has been associated with hypospadias in humans, supported by direct evidence from rodent studies (5).

Early penis development relies on steroid hormone signaling. During the fetal masculinization programming window (MPW; gestational days (GD) 16–20 in rats) (6), a surge in testosterone production by the testes prompts the bipotential genital tubercle (GT) to differentiate into a penis, while much lower testosterone levels in females lead to clitoris formation. In male fetuses, androgen receptor (AR) signaling in the GT is thought to orchestrate several signaling pathways that drive GT growth and penile structure differentiation, including urethral formation (7). This critical role of AR signaling in penis differentiation explains why developmental exposure to various anti-androgenic chemicals can cause hypospadias in rodent offspring, as seen with different AR antagonists (8, 9).

Although androgens are thought to be the primary drivers of fetal GT masculinization, estrogens may also influence penis development. Estrogen receptors (ERs) are expressed in the GT of both sexes during critical developmental stages (10, 11), and estrogenic chemicals have been linked to hypospadias in humans (12, 13), as well as in rats and mice (14, 15). Moreover, male mice with knockout of the genes encoding either ERα (*Esr1*) or aromatase (*Cyp19a1*) exhibit mild hypospadias (16, 17). The exact role of estrogen signaling in penis development remains poorly characterized, but it seems that it is the balance between androgen and estrogen signaling in the GT that ensures proper masculinization (18). In this scenario, it can be speculated that EDCs with one mode of action, for example anti-androgens, can also disrupt the other hormone pathways, in this case the estrogenic signaling pathway. In support of this, Draskau et al. (19) recently showed that the fungicide triticonazole, which has anti-androgenic properties both *in vitro* and *in vivo* (20), affected genes downstream of both AR and ER in the GT of *in utero* exposed male rats at GD17 and GD21 (19).

Understanding the molecular mechanisms linking an EDC stressor event, such as AR antagonism, to hypospadias - including its influence on other hormone pathways - can help delineate the causal toxicity pathways for EDC-induced hypospadias. In turn, detailed molecular toxicity pathways can potentially enhance the sensitivity of chemical test methods for EDC-induced effects (21). Currently, rats are often used in chemical toxicity testing, but penis development is better described in mice, highlighting a gap between developmental and toxicological research. In this study, we investigated the influence of AR antagonism on the estrogen pathway, hypothesizing that *Esr1* may be influenced transcriptionally or spatially by disruption to the androgen signaling pathway. To create a timeline of *Ar* and *Esr1* expression in the developing male rat GT, we first characterized the spatiotemporal expression patterns of both *Ar* and *Esr1* between GD15 and GD21. Subsequently, we induced hypospadias in rats by gestational exposure using the prostate cancer drug and potent AR antagonist flutamide as model chemical, with the aim to investigate how anti-androgenic EDCs may impact penis development by affecting both AR and ER signaling.

## 2 Materials and methods

### 2.1 Animals

Animal experiments were carried out at our local animal facility, with ethical approval from the national Animal Experiments Inspectorate (license number, 2020-15-0201-00539) and overseen by the local Animal Welfare Committee. The animal studies were carried out in accordance with the EU Directive, 2010/63 for the protection of animals used for scientific purposes.

Time-mated Sprague-Dawley rats (Crl: CD(SD), RRID: RGD_734476), 8–9 weeks (210–260 g; Charles River Laboratories, Sulzfeld, Germany) were used, with gestational day (GD) 1 defined as the day following overnight mating. All animals were housed in High Temperature polysulfone (H-TempTM) cages (Tecniplast, Buguggiate, Italy) with wood chip bedding, nesting material, and a Tapvei wooden shelter (Brogaarden, Lynge, Denmark). They were kept in a controlled environment with 12 h light/dark cycles, 55 ± 10% humidity, 22 ± 5 °C temperature, and 75 air changes per hour. The animals were fed a standard soy and alfalfa-free diet based on Altromin, 1314 (Altromin GmbH, Lage, Germany) and provided tap water in bisphenol A-free bottles (polyphenylsulfone 400 ml, 84ACBT0402PFS, Ultem, SCANBUR, Karlslunde, Denmark) *ad libitum*. All dams were housed in pairs until GD17 and single-housed hereafter until the end of the experiment.

### 2.2 Characterization of normal rat GT anatomy and development

Rat fetuses were collected daily from time-mated control dams at developmental stages GD15 to GD21 (n=1 dam per stage for morphological characterization by H&E stain, and n=3 dams per stage for RNAscope analyses). Dams were sedated in CO_2_/O_2_, decapitated, and fetuses were collected. The sex of the fetus was determined by gonadal inspection under a stereomicroscope (GD16, 17, 18, 20, and 21) or by genotyping of fetal rat tail for the *SRY* gene (GD15, and GD19). Whole fetuses or lower bodies were then fixed in 10% formalin, and later dehydrated in an Excelsior AS tissue processor (Axlab, Vedbaek, Denmark) and embedded on their side in paraffin.

### 2.3 Rat toxicity studies

**Figure 1:**
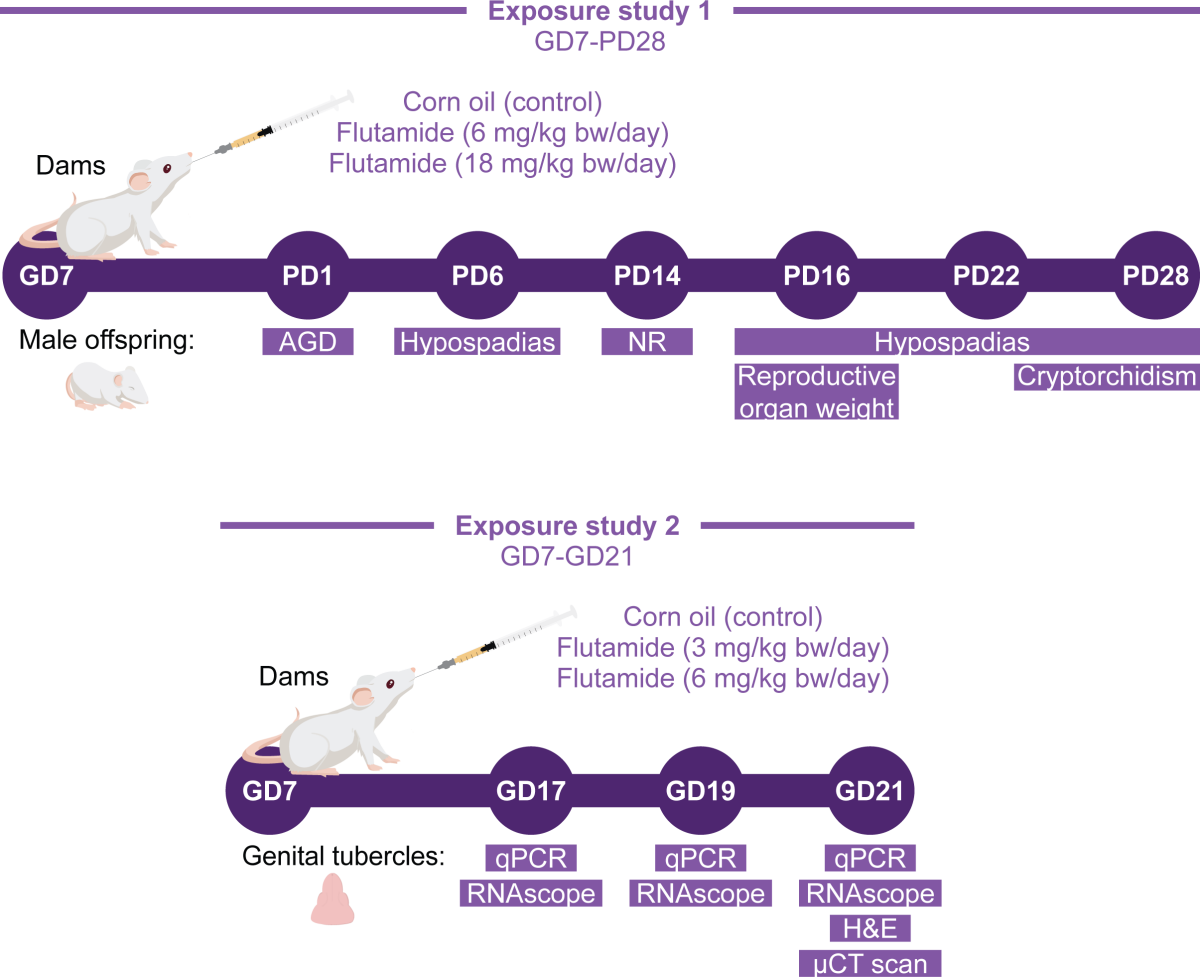
Study design for rat toxicity studies. Two exposure studies with flutamide were conducted. In exposure study 1, rats were exposed perinatally (*in utero* and during lactation) to 0, 6, or 18 mg/kg bw/day flutamide, and the postnatal phenotype of male offspring was evaluated. In exposure study 2, rats were exposed *in utero* to 0, 3, or 6 mg/kg bw/day flutamide and collected at either GD17, GD19, or GD21 for morphological and molecular analyses of the GTs.

Flutamide (>99% purity, CAS # 13311-84-7) was purchased from Merck Life Science (cat. # F9397, lot # MKCL6929). Corn oil (cat. # C8267; Merck Life Science, Søborg, Denmark) was used as vehicle. The doses were determined based on previous rat toxicity studies using flutamide as a model chemical and reporting on hypospadias (22, 23). The flutamide solutions were stored at room temperature, protected from light, and under constant stirring during the experiment. The study design is illustrated in **Figure 1**. For exposure study 1, postnatal examinations, time-mated dams were delivered to the animal facilities on GD3. The day after arrival, dams were weighed and semi-randomly assigned to three exposure groups with similar body weight (bw) distributions: 0, 6, or 18 mg/kg bw/day flutamide (n=11–12 dams per group). Flutamide or vehicle (corn oil) was administered by oral gavage, using a dosing volume of 2 ml/kg bw, from GD7 to pup day (PD) 28 (with PD1=GD23), excluding the day of birth. Rat offspring were sexed and weighed the day after birth. This procedure was repeated on PD 6, 14, 22, and 28. On PD1, anogenital distance (AGD) was measured using a stereomicroscope with a micrometer eyepiece. On PD14, the number of nipples was registered in all live rats. AGD and nipples were registered by a trained technician blinded with respect to exposure group. Testicular descent, also blinded to exposure group, was evaluated in all male rats from PD14 until PD28. The day of testicular descend was registered, and rats without full testicular descent at PD28 were registered as either uni- or bilateral cryptorchid. One or two pups per litter, randomly selected, were dissected on PD6, 16, 22, and 28. On all dissection days, the selected rats were weighed and decapitated, under CO_2_/O_2_ anesthesia for the three latter ages. The external genitalia of male offspring selected for dissection were inspected under a stereomicroscope by a technician blinded with respect to exposure group, and the presence of genital malformations was registered. A scoring system was used on PD28 to classify the severity of hypospadias and genital feminization: Score 0: No malformations (normal penis with urethral opening at the tip). Score 1: Mild hypospadias (the urethral opening is placed on ventral side of penis at the distal end). Score 2: Moderate hypospadias (the urethral opening is placed approximately on the middle along the length of the penis). Score 3: Severe hypospadias (the urethral opening is placed at the base of the penis on the ventral side). Score 4: Feminized genitalia (the external genitalia appear completely feminized).

On all designated dissection days, the penises were isolated and weighed. On PD16, the testicles, epididymis, ventral prostate, seminal vesicles, and levator ani muscle were isolated and weighed. On PD28, the dams were sedated and killed by decapitation under CO_2_/O_2_ anesthesia. The number of implantations in the dams were counted, and their liver isolated and weighed.

For exposure study 2, prenatal examinations, time-mated dams were delivered to the animal facilities on GD4. The day after arrival, dams were weighed and randomly assigned to one of three exposure groups with similar bw distributions: 0, 3, or 6 mg/kg bw/day flutamide. Flutamide or vehicle (corn oil) was administered by oral gavage from GD7 until necropsy at GD17 (n=4-6), GD19 (n=4-7), and GD21 (n=7-8). On the day of necropsy, dams were exposed to flutamide 1 h ±15 min prior to sedation in CO_2_/O_2_ and subsequent decapitation. The number of implantations and fetuses were recorded. Fetuses were isolated, and their sex was determined by visual inspection of gonads during dissection under a stereomicroscope. On GD21, the fetuses were also weighed, and AGD was measured as described above. At all stages, lower bodies or whole fetuses were fixed in 10% formalin, or the GT was isolated and placed in RNAlater and stored at -80 °C. Formalin-fixed lower bodies and full bodies were dehydrated in an Excelsior AS tissue processor (Axlab) and embedded on their side in paraffin.

### 2.4 Micro-CT scans

Micro-CT scanning was performed on paraffin-embedded fetuses from GD21 with a Phoenix Nanotom M (Waygate Technologies), operated using XS Control and Phoenix Datos|x acquisition and reconstruction software. Samples (n=3 fetuses from three different litters per group) were scanned for 10 min at 4-6 µm resolution collecting 1199 projections through a full 360-degree rotation. An X-ray tube voltage of 50 kV and current of 300 microAmp was used with a CVD coated tungsten target. Reconstructed volume data were processed using Avizo3D Pro (Thermo Fisher Scientific), reorienting specimens to common reference planes for transverse, sagittal and coronal cross-sections through the data. For 3D renders, the fetuses’ soft tissue was segmented from the µCT scan data using Avizo’s interactive thresholding tool to select slightly denser epithelial tissue and filling this in to create a sample mask. Animations illustrating the sagittal cross-section through the 3D render and in 2D are shown in **Supplementary Material** (see **Supplementary Data Sheet 1**). To highlight features, surface meshes of sample masks were generated in Avizo for ray-traced rendering.

### 2.5 RNA extraction and RT-qPCR

Total RNA was extracted from GD17 and GD19 GTs using the RNeasy Micro kit (cat # 74004, Qiagen, Hilden, Germany) and from GD21 GTs using the RNeasy Mini kit (cat # 74106, Qiagen). The sample sizes for each stage were nine for control males, six for flutamide 3 mg/kg bw/day males, ten for flutamide 6 mg/kg bw/day males, and six for control females. In some cases, multiple (at max three) fetuses per litter were used for RNA extraction. Extractions were performed according to manufacturer’s protocol, including treatment with DNase (cat # 79254, Qiagen). cDNA was synthesized from 350 ng total RNA using the Omniscript RT kit (cat # 205113, Qiagen) and Random Primer Mix (cat # S1330S, New England Biolabs, Ipswich, MA, USA). For RT-qPCR, 3 µL 1:20 diluted cDNA was added to TaqMan™ Fast Universal PCR Master Mix (2X), no AmpErase™ UNG (cat # 4352042, Applied Biosystems^®^, Waltham, MA, USA) and a TaqMan™ Gene Expression Assay in a 384-well plate. A list of the gene assays is provided in **Supplementary Table 1**. The RT-qPCR reactions were run on a QuantStudio 7 Flex Real-Time PCR system (Applied Biosystems^®^). Relative gene expression was calculated using the comparative Ct method and the geometric mean of two references genes *Rps18* and *Sdha* (24).

### 2.6 Histology and RNAscope^®^

Lower section of bodies and whole bodies were sagittally sectioned at 5 µm thickness. Tissues were deparaffinized by heat treatment (60 °C for 30–60 min) and petroleum wash and subsequently rehydrated by ethanol treatment in decreasing strengths. For structural analyses (one fetus per examination stage), sections were stained with hematoxylin and eosin (H&E), dehydrated through an ethanol series, and mounted using Eukitt^®^ Quick-hardening Mounting Medium (cat # 03989, Merck Life science). Expression of *Ar, Esr1* RNA molecules in the GT (minimum three litters per staining, one fetus per litter) was analyzed on the deparaffinized sections using the RNAscope^®^ *in situ* hybridization kit (cat # 322350 Bio-Techne, Dublin, Ireland) according to manufacturer’s instructions. Briefly, endogenous peroxidase activity was blocked with hydrogen peroxidase treatment, and sections were boiled in RNAscope^®^ Target Retrieval Buffer. A hydrophobic barrier pen (cat # 310018, Bio-Techne) was used to draw a barrier around each section, and the sections were left to dry overnight. The next day, sections were protease-treated for 15 min, and the probe of interest hybridized for 2 h at 40 °C. A negative and a positive control probe were also included. The probe signal was amplified by addition of 6 RNAscope^®^ amplification probes, and signal was detected by RNAscope^®^ Fast Red chromogenic substrate. The sections were counterstained in hematoxylin, dried at 60 °C for 30 min, and mounted in VectaMount^®^ Mounting Medium (cat # 321584, Bio-Techne). A list of the probes used is provided in **Supplementary Table 1**. Sections were scanned using the PANNORAMIC Midi II slide scanner (3D Histech, Budapest, Hungary), using a 40x objective with 116x Native output resolution. Images were processed in CaseViewer version 2.4. For changes between groups in exposure study 2, semi-quantitative analyses were made in ImageJ version 2.14.0 using contrast thresholding.

### 2.7 Statistical analysis

All statistics were performed in R version 4.2.3, except for nipple/areola counts. AGD index (AGDi) was calculated as AGD/^3^√body weight. AGD, AGDi, and body weights were analyzed using a linear least square means mixed-effects model (lme4 (25), RRID: SCR_015654) with litter as a random and nested factor (except for PD16 and PD28 body weights, for which only 1 animal per litter was weighed). For birth weight, number of pups was included as a co-variate. P-values for exposure were obtained by Dunnett’s *post hoc* analysis. Dam and pregnancy data were analyzed by linear least square means regression and Dunnett’s *post hoc* analysis, with the exception for post-implantation and perinatal loss, for which the Kruskal-Wallis test and Dunn’s *post hoc* test were used. Reproductive organ weights were analyzed by linear least square means regression and Dunnett’s *post hoc* test, using body weight as a co-variate for the organ weights. qPCR data was analyzed using least square means regression and Dunnett’s *post hoc* analysis. For organs smaller than the detection limit of the scale (3 mg) the weight was imputed to 3 mg. In cases of organ agenesis (this was seen for prostate and seminal vesicles), the weight of the organ was set to 0 mg. Nipple retention was analyzed in SAS by generalized estimating equations (GEE) using a binomial model with 13 as the maximal number of nipples and using litter as the cluster. “Exchangeable” was used as the correlation structure, and significance level was assessed by a Hommel procedure. Data are presented as mean ± SD or boxplot with min, max, and 25^th^, 50^th^, and 75^th^ percentiles, *P<0.05, **P<0.01, ***P<0.001.

## 3 Results

### 3.1 Normal anatomy and development of the male rat GT from GD15-GD21

**Figure 2:**
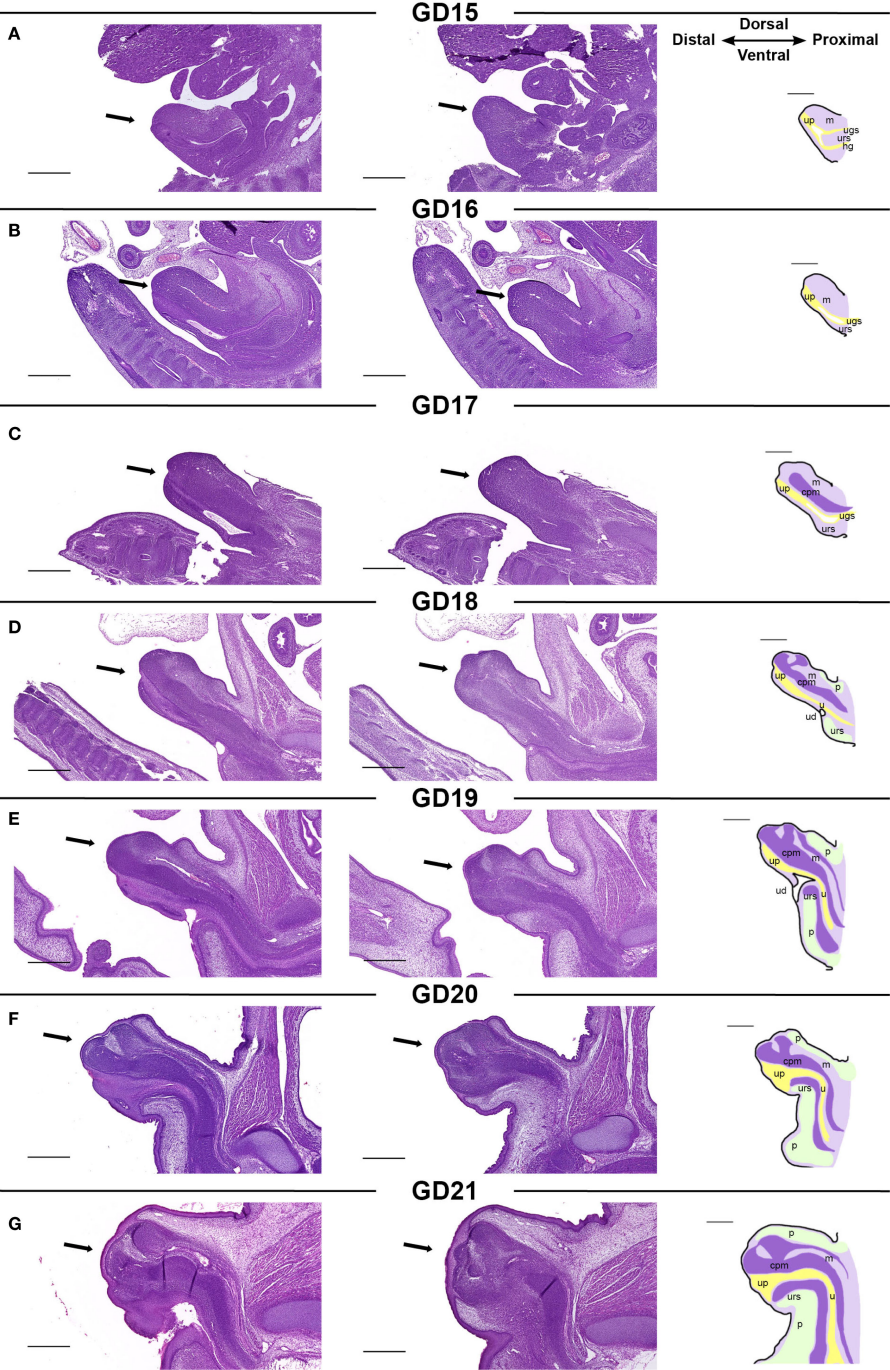
**(A-G)** Morphology of the developing male rat genital tubercle from GD15 to GD21. Sagittal sections of GTs (indicated by arrows) at each developmental stage stained with H&E. The two histological pictures from one developmental stage are from the same sample, 70 µm apart. The specific structures visible in a mid-sagittal section of the GT are indicated on sketches in the right column. cpm, central penile mesenchyme; h, hindgut; m, mesenchyme; p, prepuce; u, urethra; ud, urorectal duct; ugs, urogenital sinus; urs, urorectal septum. Scale bars: 500 µm.

To gain a comprehensive overview of each stage of rat GT development, we mapped the development of the male rat GT between GD15–21 by histological assessments (**Figure 2**). We combined terminologies from previous descriptions of rat (26–28) and mouse (29) GT development at selected time points, utilizing these to map the timing and appearance of specific structures of the rat GT. The GT arises from two swellings that merge around the cloaca, which by GD15 has separated into the urogenital sinus and hindgut by the urorectal septum. At this stage, a urethral plate has formed distally on the ventral side, and this will later generate the urethra (**Figure 2A**). At GD15 and GD16, the GT grows, and the dorsal mesenchyme expands (**Figure 2B**). From GD17, a dense, central penile mesenchyme is visible in the center of the GT, which continues to expand throughout fetal development to form most of the glans penis by GD21 (**Figures 2C-G**). At GD18, the proximal urethra has formed, and the urorectal duct is seen as an opening of the urethra. In males, the urorectal duct is closed by the differentiation of the proximal part of the urethra (**Figure 2D**). This differentiation is largely driven by the urorectal septum, which invades the urethral plate from ~GD18 to form an internalized urethra in the glans (**Figures 2D-G**). In parallel, prepuce is visible on the dorsal side of the GT from GD18 and on the ventral side from GD19 (**Figures 2D, E**), and the prepuce gradually envelops the glans in a proximal to distal direction from GD18 to GD21, fusing at the ventral midline (**Figures 2D-G**).

### 3.2 Androgen receptor is expressed throughout the genital tubercle during development

**Figure 3:**
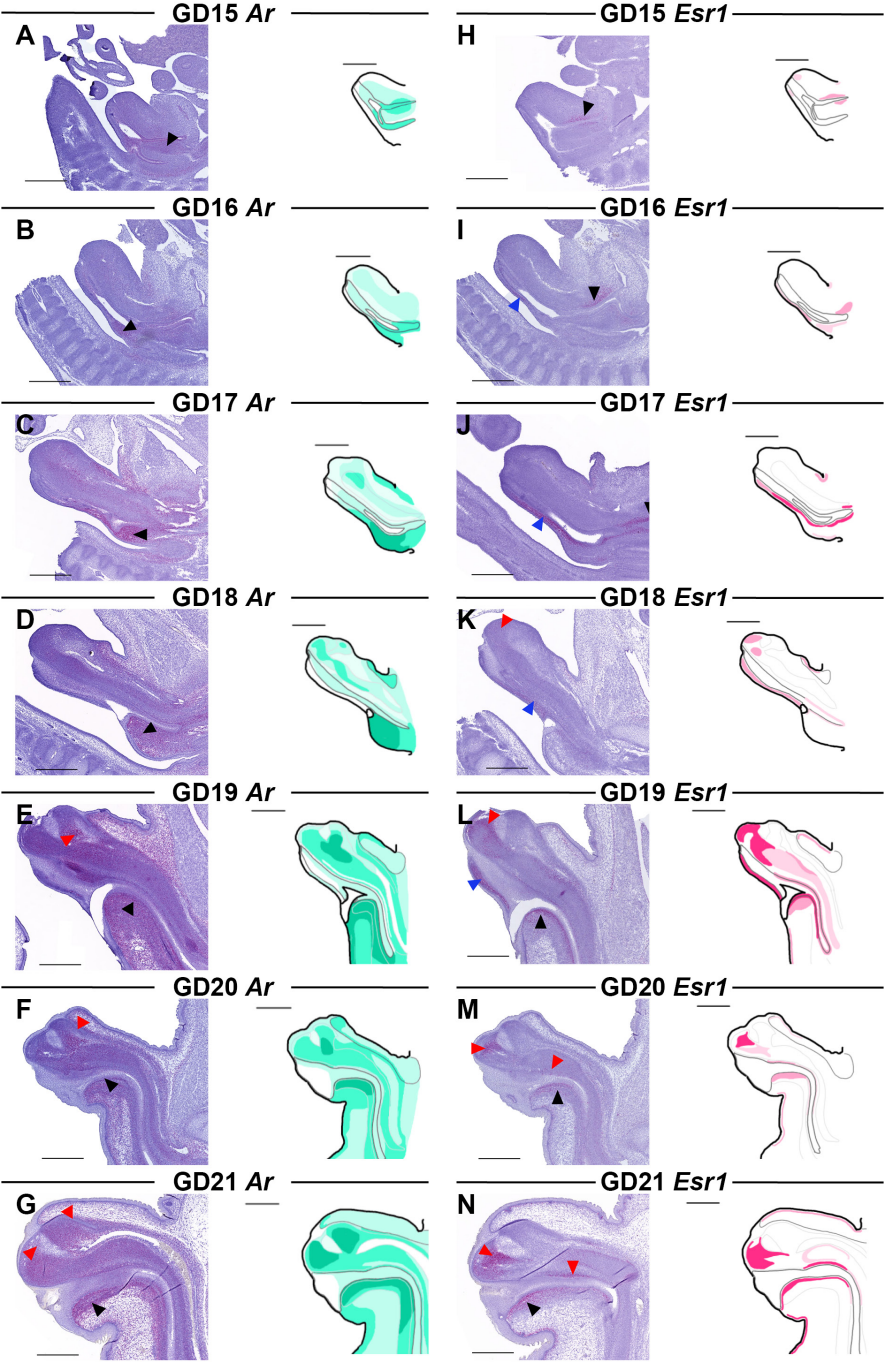
*Ar* and *Esr1* expression in the male rat GT from GD15-21. Sagittal sections of GTs at each developmental stage stained for *Ar* **(A-G)** and *Esr1* **(H-N)** using RNAscope^®^ *in situ* hybridization. The areas of *Ar* and *Esr1* expression are indicated in drawings by graded coloring of green and pink, respectively. Specific regions of higher expression highlighted by arrowheads in the images as specified in the text. Representative images of 3 replicates, scale bars: 500 µm.

Using *in situ* RNA hybridization (RNAscope^®^), we mapped the transcriptional distribution of *Ar* and *Esr1* in the GT from GD15-21 (**Figure 3**). *Ar* is expressed diffusely throughout the GT at all developmental stages, increasing as development progresses. Additionally, there are certain regions of the GT with consistently higher *Ar* expression, particularly in the urorectal septum and central penile mesenchyme. At GD15 and GD16, when *Ar* expression is generally lower than at later stages, the urorectal septum is the region with highest *Ar* expression (**Figures 3A, B**, black arrowheads). At GD17 and GD18, the entire cell-dense urorectal septum is expressing *Ar* (**Figures 3C, D**, black arrowheads) but as it grows into the urethral plate, *Ar*-positive cells are most densely located just ventral to the urethra (**Figures 3E-G**, black arrowheads). The central penile mesenchyme is seen from GD17, at which point it has a slightly higher *Ar* expression than the surrounding mesenchyme (**Figure 3C**). At GD19, the central penile mesenchyme contains localized areas of high *Ar* expression in the distal part of the GT (**Figure 3E**, red arrowhead), which persist through GD20 and GD21 (**Figures 3F, G**, red arrowheads).

### 3.3 Estrogen receptor is expressed in specific areas of the genital tubercle during development

*Esr1* expression is limited to specific areas of the GT during development. At GD15-16, *Esr1* expression is very weak, and only localized to small areas, mainly around the urogenital sinus where expression peaks at GD17 (**Figures 3H-J**, black arrowheads). Low *Esr1* expression is also seen at the ventral side along the urethral plate at GD16 (**Figure 3I**, blue arrowhead), which continues from GD17-19 (**Figures 3J-L**, blue arrowheads). From GD19, *Esr1* is expressed in the urorectal septum, specifically in mesenchymal cells just ventral to the urethra (**Figures 3L-N**, black arrowheads). In the central penile mesenchyme, *Esr1* is first seen at GD18, although weakly and restricted to the very distal end (**Figure 3K**, red arrowhead). At GD19, more of the central penile mesenchyme expresses *Esr1* with the highest expression still being at the distal end (**Figure 3L**, red arrowhead). At GD20 and GD21, *Esr1* is again mainly expressed in the distal tip of the glans central penile mesenchyme, as well as on the dorsal side of the urethra (**Figures 3M, N**, red arrowheads). Lastly, when the prepuce starts appearing at GD18, a thin layer of *Esr1-*positive cells lies beneath the epithelium in both the dorsal and ventral developing prepuce (**Figures 3K-N**).

### 3.4 Validation of the flutamide-induced hypospadias model

Having mapped the expression patterns of *Ar* and *Er* during normal penis development in rats, we next investigated how the expression was affected by an EDC inducing hypospadias. To have a model of hypospadias, we utilized the potent AR antagonist and pharmaceutical flutamide, which has previously been used as a model chemical to induce hypospadias in rats (9, 28). We first validated the hypospadias model by perinatal (GD7-PD28) exposure of rats, via the dams, to flutamide in doses of either 6 or 18 mg/kg bw/day. Dam, pregnancy, and offspring data, including reproductive parameters in male offspring, are provided in **Supplementary Tables 2**-**4**. Genital malformations were visible in the flutamide-exposed male rats as early as PD6 (**Supplementary Table 3**). At PD28, male fetuses exposed to 6 mg/kg bw/day flutamide (one per litter) had either moderate or severe hypospadias, while 10/11 of the male rats exposed to 18 mg/kg bw/day flutamide had feminized external genitalia with the last one in this group having severe hypospadias (**Table 1**).

**Table 1:** Genital malformations at PD28 in male rats exposed perinatally to flutamide.

Male offspring PD28			
	Control	Flutamide (6 mg/kg bw/day)	Flutamide (18 mg/kg bw/day)
Number of litters	11	11	11
Genital malformation score PD28			
No malformation	11	0	0
Mild hypospadias	0	0	0
Moderate hypospadias	0	4	0
Severe hypospadias	0	7	1
Feminized phenotype	0	0	10

Rats were exposed *in utero* and throughout lactation to flutamide (0, 6, or 18 mg/kg bw/day). One male rat per litter, randomly chosen, was inspected under a stereomicroscope and the external genitalia were scored by a technician blinded with respect to exposure group.

### 3.5 Adverse reproductive effects of flutamide are visible prenatally in male rats

We next explored how the hypospadias phenotype develops prenatally. Due to the feminized phenotype caused by 18 mg/kg bw/day flutamide, we replaced this dose with a lower dose of flutamide (3 mg/kg bw/day). Pregnant rats were thus administered flutamide (0, 3, or 6 mg/kg bw/day) from GD7 until necropsy at either GD17, GD19, or GD21. Dam and pregnancy data for litters necropsied at GD21 are provided in **Supplementary Table 5**. In the absence of postnatal data for 3 mg/kg bw/day flutamide, we assessed the adverse reproductive effects of flutamide prenatally by examining the GD21 fetuses.

**Figure 4:**
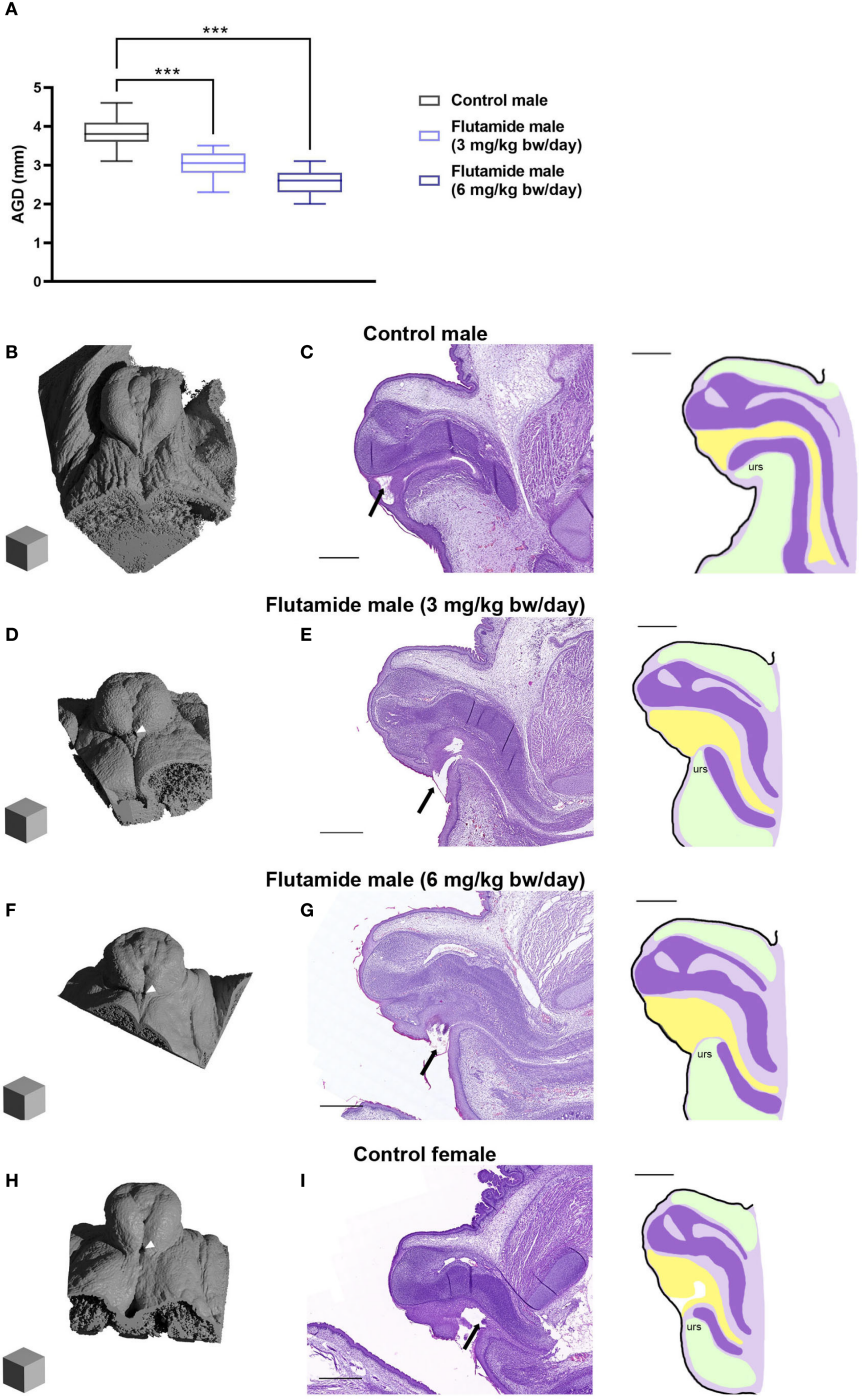
Adverse reproductive effects of flutamide in male rats at GD21. Rats were exposed *in utero* to flutamide at 0, 3 or 6 mg/kg bw/day from GD7 to GD21. **(A)** Anogenital distance (AGD) measured at GD21. Data shown as box plots with min, max, 25^th^, 50^th^ and 75^th^ percentiles, n=7–8 litters, ***p<0.001 by a linear least square means mixed-effects model with litter as a random and nested factor and Dunnett’s *post hoc* test. See also **Supplementary Table 6**. **(B, D, F, H)** Ray-traced renders of µCT scans of GD21 GTs (Blender 4.2 LTS) Scalecubes: (500 µm)^3^. White arrowheads indicate the proximal opening at the base of the GT. **(C, E, G, I)** H&E stain of GD21 GTs sectioned sagittal with corresponding sketches on the right, urs: urorectal septum (see **Figure 1** for more specifications). Arrows indicate the urethral opening. Scalebars: 500 µm. See also **Supplementary Videos 1**-**4**.

At GD21, AGD was shorter in male fetuses in both flutamide exposure groups compared to control male fetuses (**Figure 4A**, 76.9 and 66.7% of control, respectively, p<0.001), with no difference in body weight (**Supplementary Table 6**). In the GT of control males, the preputial folds were closing at the ventral midline with a single urethral opening near the distal tip of the GT (**Figures 4B, C**). In flutamide-exposed males, the folds were likewise closing in at the midline but there was an opening in the tissue towards the base of the GT (**Figures 4D, F** arrowheads); histologically, it was also clear that the urethral opening was more proximally located (**Figures 4E, G**, arrows). This appeared to be caused, at least partly, by underdevelopment of the urorectal septum and fusion of the ventral prepuce. Thus, both doses of flutamide disrupted masculinization in the fetuses, with hypospadias phenotypes presenting already prenatally. In general, the phenotype in the higher flutamide dose group (6 mg/kg bw/day) was more severe than the lower flutamide dose group (3 mg/kg bw/day). The morphological and histological phenotypes observed in flutamide-exposed GTs largely resembled the female GT at GD21 (**Figures 4H, I**). Animations of µCT scans are available in **Supplementary Files** (see **Supplementary Data Sheet 1**).

### 3.6 *In utero* exposure to flutamide alters *Esr1* expression in the fetal genital tubercle

**Figure 5:**
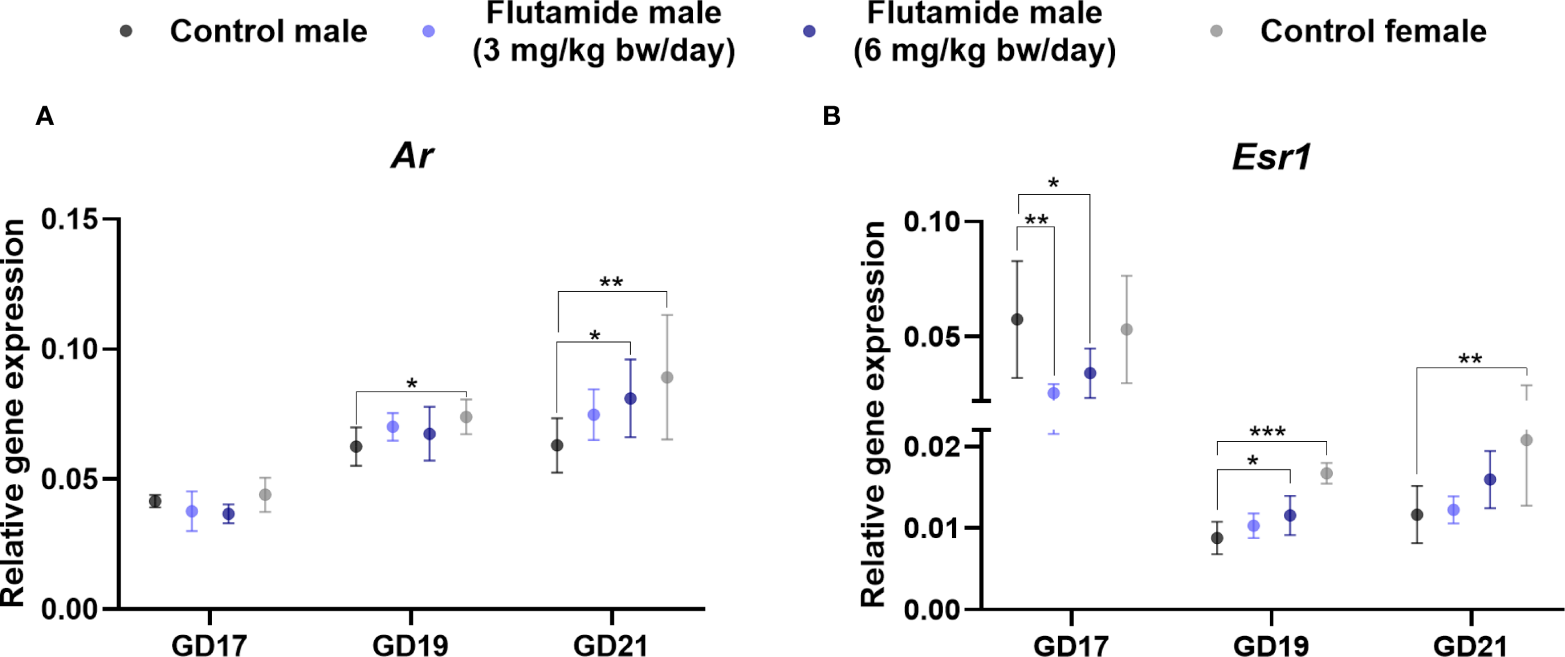
Gene expression of *Ar* **(A)** and *Esr1* **(B)** in genital tubercles exposed to flutamide. Rats were exposed in utero to flutamide at 0, 3 or 6 mg/kg bw/day from GD7 to GD17, GD19, or GD21. Gene expression levels were related to the geometric mean of expression of two housekeeping genes Rps18 and Sdha. Data shown as mean ± SD, n=6-10, p-values by least square means regression and Dunnett’s post hoc analysis. *p<0.05, **p<0.01, ***p<0.001.

To decipher how exposure to anti-androgenic substances may alter the hormone signaling pathways in the developing GT, we investigated gene expression levels of *Ar* and *Esr1* at GD17, 19, and 21 by RT-qPCR, comparing control males to flutamide-exposed males and control females (**Figure 5**). Exposure to 3 mg/kg bw/day flutamide did not change expression of *Ar* in the GT, but 6 mg/kg bw/day increased *Ar* expression at GD21 (**Figure 5A**, p=0.04). Moreover, *Ar* expression was higher in control females at GD19 and GD21 compared to control males (**Figure 5A**, p=0.03 and p=0.008, respectively). For *Esr1*, both doses of flutamide decreased its expression in the GT at GD17 (**Figure 5B**, p=0.008, and p=0.04 for 3 and 6 mg/kg bw/day, respectively), while on GD19, the expression of *Esr1* was increased in flutamide (6 mg/kg bw/day) males (**Figure 5B**, p=0.01). There was no difference in *Esr1* expression between flutamide-exposed and control males at GD21 (**Figure 5B**). *Esr1* expression was higher in control females at both GD19 and GD21 compared to control males (**Figure 5B**, p<0.001, and p=0.002, respectively).

### 3.7 Expression of *Ar* in the ventral region of the genital tubercle is sexually dimorphic and increased by flutamide exposure

**Figure 6:**
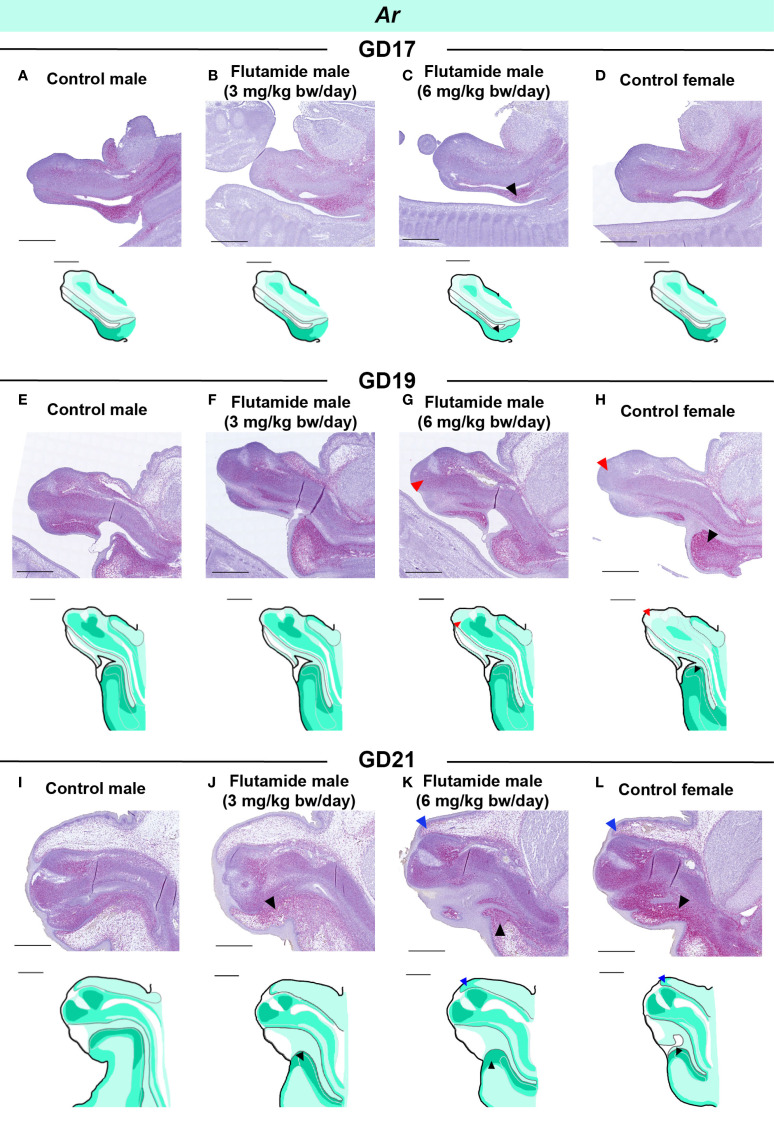
Expression of *Ar* in genital tubercles exposed to flutamide. Rats were exposed in utero to flutamide at 0, 3 or 6 mg/kg bw/day from GD7 to GD17 **(A-D)**, GD19 (E-H), or GD21 **(I-L)**. Sagittal sections of GTs at each developmental stage stained for Ar using RNAscope® in situ hybridization. Areas of altered expression by flutamide or in females compared to control males are indicated by arrowheads (colors specified in text) and in drawings by graded coloring. Representative images of 3 replicates, scale bars: 500 μm. See also **Supplementary Figure 1**.

Both *Ar* and *Esr1* expression was, at some stage during development, affected by flutamide exposure as observed by RT-qPCR. We thus examined the spatial expression patterns of *Ar* and *Esr1* in the GT of flutamide-exposed fetuses by RNAscope (summary of changes in **Supplementary Figure 1**). We performed semi-quantitative analyses on areas of the GT to assess any regional changes in expression (**Supplementary Figures 2**, **3**). Despite no quantitative difference in *Ar* levels at GD17 or GD19 compared to controls (**Figure 5A**), males exposed to 6 mg/kg bw/day flutamide appeared to have lower *Ar* expression in the urorectal septum at GD17 and in the central penile mesenchyme at GD19 (**Figures 6C, G**, black and red arrowheads, respectively). At GD21, *Ar* expression in the ventral region of the GT, including the urorectal septum and ventral prepuce, was increased in males in both flutamide groups (**Figures 6J, K**, black arrowheads). The higher *Ar* levels at GD19 and GD21 in females (**Figure 5A**) were likewise mainly due to higher expression in the urorectal septum and ventral prepuce (**Figures 6H, L**, black arrowheads), although at GD19 concomitant with lower *Ar* expression in the distal central clitoral mesenchyme (**Figure 6H**, red arrowhead). Moreover, a subtle increase in *Ar* expression in cells beneath the epithelium in the dorsal prepuce was evident at GD21 both in females and in males exposed to 6 mg/kg bw/day flutamide (**Figures 6K, L**, blue arrowheads).

### 3.8 Flutamide increases expression of *Esr1* in distinct regions of the GT at GD19 and GD21

**Figure 7:**
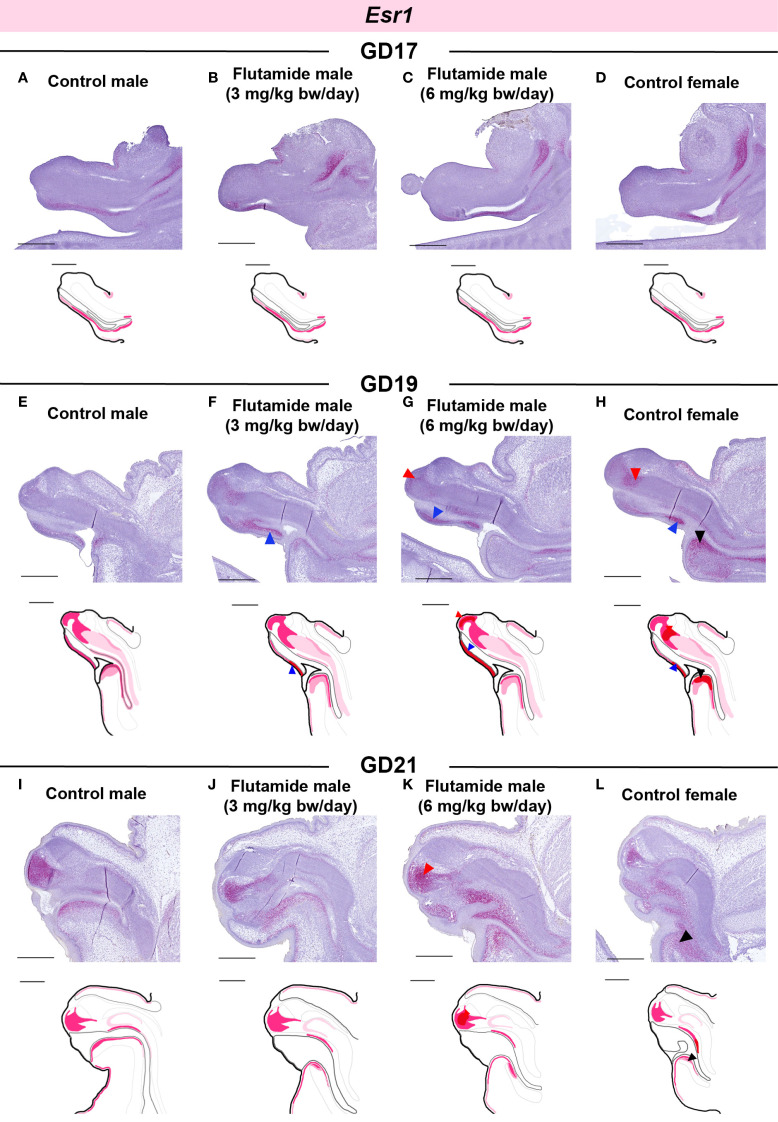
Expression of *Esr1* in genital tubercles exposed to flutamide. Rats were exposed in utero to flutamide at 0, 3 or 6 mg/kg bw/day from GD7 to GD17 **(A-D)**, GD19 **(E-H)**, or GD21 **(I-L)**. Sagittal sections of GTs at each developmental stage stained for *Esr1* using RNAscope® *in situ* hybridization. Areas of altered expression by flutamide or in females compared to control males are indicated by arrowheads (colors specified in text) and in drawings by graded coloring. Representative images of 3 replicates, scale bars: 500 µm. Se also **Supplementary Figure 1**.

In GD17 GTs, there were no noticeable differences between the four groups in the spatial expression of *Esr1* (**Figures 7A-D**), despite lower levels being measured in flutamide-exposed males by RT-qPCR (**Figure 5B**). *Esr1* expression was increased in the ventral mesenchyme of both flutamide groups at GD19 (**Figures 7F, G**, blue arrowheads) and in the central penile mesenchyme of the 6 mg/kg bw/day flutamide group at GD19 and GD21 (**Figures 7G, K**, red arrowheads). Noticeably, flutamide did not induce ectopic expression of *Esr1* in new areas of the GT, but instead increased levels in the distinct *Esr1*-positive areas. In females, *Esr1* expression was higher compared to control males at GD19 in the central mesenchyme, ventral mesenchyme, and urorectal septum (**Figure 7H**, red, blue, and black arrowheads, respectively), and at GD21 in the urorectal septum only (**Figure 7L**, black arrowhead).

## 4 Discussion

Fetal masculinization is strongly hormone-dependent, and endocrine disruptions can lead to congenital defects. Fetal exposure to various anti-androgenic chemicals induces genital malformations, such as hypospadias, in male rodents (8, 9, 30, 31), as also demonstrated in this study with the AR antagonist flutamide. While the impacts of anti-androgenic EDCs on penis differentiation are well-established, their effects on estrogen signaling are less well characterized, as are the close interactions between these two signaling pathways during both normal and stressed conditions. Given the susceptibility to develop hypospadias from exposure to both anti-androgenic and estrogenic substances, it is important to characterize this mechanism in more detail. This study supports the hypothesis that estrogenic and androgenic signaling in the GT are closely connected and, importantly, that disruption of one signaling pathway (in this case, AR) affects the other (in this case, *Esr1*). Exposure to the AR antagonist flutamide alters *Esr1* expression through spatial changes in *Esr1*, specifically in tissue structures critical for GT growth and differentiation.

Although many rat toxicity studies report hypospadias as an adverse outcome, most of the current understanding of the molecular mechanism underlying penis differentiation comes from mouse studies. Therefore, one objective of our study was to map the development of the male rat GT through histological assessments to establish a foundation for defining chemical effects in rat toxicity studies. We found that *Esr1* was expressed at all stages of development between GD15 and GD21, with expression restricted to certain areas in the male GT. This contrasts with the broad expression of *Ar* throughout the GT. In mice, single-cell sequencing has revealed distinct cell populations in the glans of the GT (10, 32), which may help explain why only a subset of cells in the glans express *Esr1.* Another region of the GT with high expression of both *Ar* and *Esr1* is the urorectal septum. This structure appears early in development, separating the cloaca into the urogenital sinus and the hindgut. Later, during the differentiation of the GT, the urorectal septum invades the urethral plate in males to form a central urethral tube (29, 33). This process of urethral plate invasion by the urorectal septum is observable in our GD15-GD21 histological timeline, where the septum gradually grows along the ventral surface of the penis until the urethral opening is located at the tip. This process is disrupted by flutamide, where at GD21, the urorectal septum has not grown as much distally, resulting in a proximally placed urethral opening.

The effect of flutamide on urorectal septum growth and urethral closure underscores the hormone dependency of the urorectal septum and the ventral side of the GT in general. In our mapping of *Ar* and *Esr1* expression throughout GT development, receptor expression increases in this area as development progresses. The urorectal septum is one of the earliest *Esr1*-expressing regions (from GD16) and continues to express *Esr1* in a well-defined region of the ventral GT. While *Ar* is expressed in both the urorectal septum and the prepuce on the ventral side of the GT, *Esr1* is mainly seen in the urorectal septum and the ventral mesenchyme next to the urethral plate. This could suggest that the urorectal septum and ventral mesenchyme depend not only on androgen signaling but also on estrogen signaling, and the two pathways may be strongly and mutually regulating each other in this specific region. This is supported by flutamide-induced alterations in *Ar* expression at GD17 (downregulation) and GD21 (upregulation) in the urorectal septum and *Esr1* in the ventral urethral plate mesenchyme, as well as by the sexual dimorphic expression of *Ar* and *Esr1* in the urorectal septum at GD19 and GD21 in control rats. As expected, most of the expression pattern changes in the GT of flutamide-exposed males resembled those in the GT of female control rats. This highlights the complexity of how the overall balance in hormone signaling within specific cell types regulates tissue differentiation.

In humans, ERα protein expression is also restricted to specific regions of the developing penis, mainly around the preputial lamina and in the canalizing urethral plate from around gestational week 15 (11, 34), which is after the MPW in humans (suggested to be gestational weeks 8-14) (6, 35). Thus, despite the region-specific nature of ERα expression in both rats and humans, there seem to be significant species differences in the timing and location of nuclear receptor expression, which could indicate differences in the receptor’s role in GT development. The absence of ERα protein in the human glans penis also contrasts with its high mRNA expression in the distal glans in the male rat GT, suggesting that ERα might play a more functionally important role in rats than in humans. Conversely, ERβ is expressed in several regions of the developing human penis, including the glans, corporal body, urethral groove, and prepuce (11, 34). *Esr2*, which encodes ERβ in rodents, is also expressed in the rat penis (36), but studies in knockout mice suggest that ERα is the functionally relevant receptor for rodent penis differentiation (16, 17, 37, 38). Some of the differences between humans and rats could be due to measurement of protein or mRNA, respectively, but overall, these differences warrant further research to establish the role of each receptor subtype in human penile development.

In this study, we used flutamide to induce hypospadias in rats by an anti-androgenic mode of action. First, we validated the model in a longer, perinatal exposure study, where flutamide reliably induced hypospadias in male offspring. In a second, prenatal study, with a shorter exposure window (GD7-GD21), we observed early signs of hypospadias already at GD21. This emphasizes that while hypospadias is mainly registered postnatally, it originates prenatally and is highly dependent on androgen signaling in the MPW. Consequently, molecular changes in the GT during the MPW are highly relevant to understanding hypospadias pathogenesis, and thus the core focus of this study. Since flutamide is a selective AR antagonist, it likely does not directly interact with ERs or other steroid hormone receptors at the used concentrations (39). Nevertheless, AR antagonism quantitatively altered *Esr1* expression at GD21. Other studies have also shown that anti-androgen and androgen exposure affects ER expression in the GT. For example, the AR antagonist vinclozolin increased fetal *Esr1* levels in male mouse GTs (40), and dibutyl phthalate (DBP), which lowers testosterone levels *in vivo*, elevated ERα protein levels in rats at PD10 (41). Exposure of female mice to testosterone reduced *Esr1* mRNA and ERα protein levels in the clitoris at PD1, while estradiol benzoate exposure in males decreased *Ar* expression in the penis at PD1 (18).

While these results align with our results in flutamide-exposed males, the literature on *Ar* expression remains inconsistent. Our study showed higher *Ar* expression in females at GD19 and GD21 and in flutamide-exposed males at GD21 compared to control males, a finding also supported by single-cell RNA sequencing of mouse GTs at E18.5 (10). This contrasts with studies reporting lower *Ar* levels in the female clitoris, which could be increased at PD1 by testosterone treatment (18). Rat toxicity studies with various anti-androgens showed lower *Ar* mRNA or AR protein levels postnatally (28, 41–45), which differ from our findings in prenatal GTs. Human hypospadias cases also show inconsistent results, with some reporting increased *ESR1* and decreased *AR* expression in foreskin samples (46), while others found decreased *ESR1* and *ESR2* in hypospadias cases (47). Overall, these data again highlight the interplay between androgen and estrogen signaling in the GT, and our study contributes by demonstrating the spatial changes that accompanies a quantitative shift in receptor expression, particularly in the urorectal septum and ventral mesenchyme. In other words, we here show that, beyond androgen and estrogen signaling affecting each other quantitatively, impacting one signaling pathway can alter the spatial expression pattern of nuclear receptors of the opposing (non-targeted) signaling pathway.

In this study, we investigated two genes (*Ar* and *Esr1*) that are crucial for maintaining sex-hormone signaling balance in the developing GT. Both were, at some point during development, differentially expressed in flutamide-exposed GTs and in female GTs compared to control males Interestingly, the transcriptional (quantitative) changes in *Ar* and *Esr1* were not always temporally consistent, which was likely attributed to different spatial changes across ages, such as for example seen in females at GD19, where *Ar* was increased in one area and decreased in another. The fact that regulation of *Ar* transcription is spatially dependent, makes interpretation of bulk-RNA results more challenging. A similar conclusion can be made for *Esr1*, where the quantitative decrease by flutamide at GD17 was not confirmed spatially. Although speculative, this discrepancy could be that the changes occurred in more peripheral areas of the GT, or the changes were in such a pattern (i.e. in several different areas), that the decrease in each area was too subtle to detect.

In general, these results also support the coupling of bulk transcriptional quantifications with spatial visualizations. As is also clear from our histological timeline, the GT is highly heterogenous, progressing through marked tissue changes over a short period of time. Thus, spatial investigations of expression are of high value in understanding where important changes occur. However, it must also be noted that because of this heterogeneity, spatial investigations are equally challenging to assess, in particular quantitatively. Two different sectioning planes are commonly used for GT assessments: coronal or sagittal, both challenging techniques with regard to the GT. In this study, we chose sagittal sectioning to gain a more comprehensive view of the length of the central GT and the urorectal septum, but neither sagittal nor coronal sectioning will provide a full picture of all structures and changes, which must be kept in mind when interpreting the results.

While *Ar* and *Esr1* likely play a role in the severe hypospadias phenotypes observed postnatally in males exposed to 6 mg/kg bw/day of flutamide, it is probable that other molecular changes are also involved. In exposure scenarios such as this study, numerous other genes are differentially expressed, as previously shown (19, 48). During development, different signaling pathways are activated at different times to exert their specific role in coordinating GT differentiation. Much is still to be uncovered about penis differentiation, in particular the hormone-sensitive period. One gene downstream of AR suspected to be involved in urethral closure is *Mafb*, a sexually dimorphic transcription factor expressed in the periurethral mesenchyme of males. Its expression is induced by testosterone in female mice and reduced in male *Ar* knockout mice, and ablation of the gene causes hypospadias (49). Other potential signaling pathways involved are the Fibroblast Growth Factor (Fgf) and Wnt-signaling pathways, which are both transcriptionally affected by antagonism of AR in mice (18), and knockout of Fgf receptor 2 causes severe hypospadias in mice (50). Apart from these examples, there are numerous genes implicated in hypospadias by their role in normal penis development (7, 10, 51), and more non-targeted analyses by single-cell sequencing will undoubtedly shed more light on how various signaling pathways are involved. These genes should be analyzed in future studies. However, given the critical roles of estrogen and androgen signaling in normal penis development, the disruption of these receptor pathways is key to understanding the effects of endocrine disruption. Our control timelines and results from flutamide-exposed rats demonstrate the complexity in hormone signaling and interactions that chemicals, including those less potent than flutamide, may disrupt. While model chemicals such as flutamide may have a single mode-of-action, environmental EDCs may often exhibit several modes-of-action, and in some instances the mechanism may not be fully characterized (52). The link between EDCs and hypospadias is well established in animals and supported by epidemiological studies in humans (5, 12, 53–56). To better understand how and when EDCs induce hypospadias, it is essential to further characterize the local interactions between AR and ER signaling axes in sex-hormone sensitive tissues, as the two pathways probably cannot be disentangled when evaluating EDC effect outcomes *in vivo*.

## 5 Conclusions

With this study, we have provided further evidence supporting the hypothesis that androgen and estrogen signaling jointly regulate sexual differentiation of the external genitalia in mammals. Exposure to the AR antagonist flutamide disrupted androgen signaling as expected, but also revealed simultaneous disruption to estrogen signaling. We contribute new knowledge by showing not only quantitative changes in *Esr1* expression in hypospadiac rat penises but also showing a spatial change in *Esr1* expression. This, along with the sexual dimorphic, and co-localized expression of *Ar* and *Esr1* in the developing urorectal septum, highlights the sex-specific hormone-responsiveness of the differentiating genital tubercle in determining penile or clitoral fate. Understanding the interplay between hormone signaling pathways is crucial for identifying the context of EDC actions and for developing new test methods for predictive toxicology aimed at replacing animal testing of chemicals.

## Data Availability

The raw data supporting the conclusions of this article will be made available by the authors, without undue reservation.

## Supplementary material

Supplementary Video 1 Control male GD21, related to **Figure 4**.

Supplementary Video 2 Control female GD21, related to **Figure 4**.

Supplementary Video 3 Flutamide (3 mg/kg bw/day) male GD21, related to **Figure 4**.

Supplementary Video 4 Flutamide (6 mg/kg bw/day) male GD21, related to **Figure 4**.
