# Supplementary material for: Androgen receptor antagonist flutamide modulates estrogen receptor alpha expression in distinct regions of the hypospadiac rat penis
Source: Front Endocrinol (Lausanne). 2025 Sep 12;16:1654965. doi: 10.3389/fendo.2025.1654965 (PMC12464887; doi:10.3389/fendo.2025.1654965)
Supplement: Supplementary file 5 [file DataSheet1.docx]

Supplementary Material

# Supplementary Figures and Tables

## Supplementary Figures


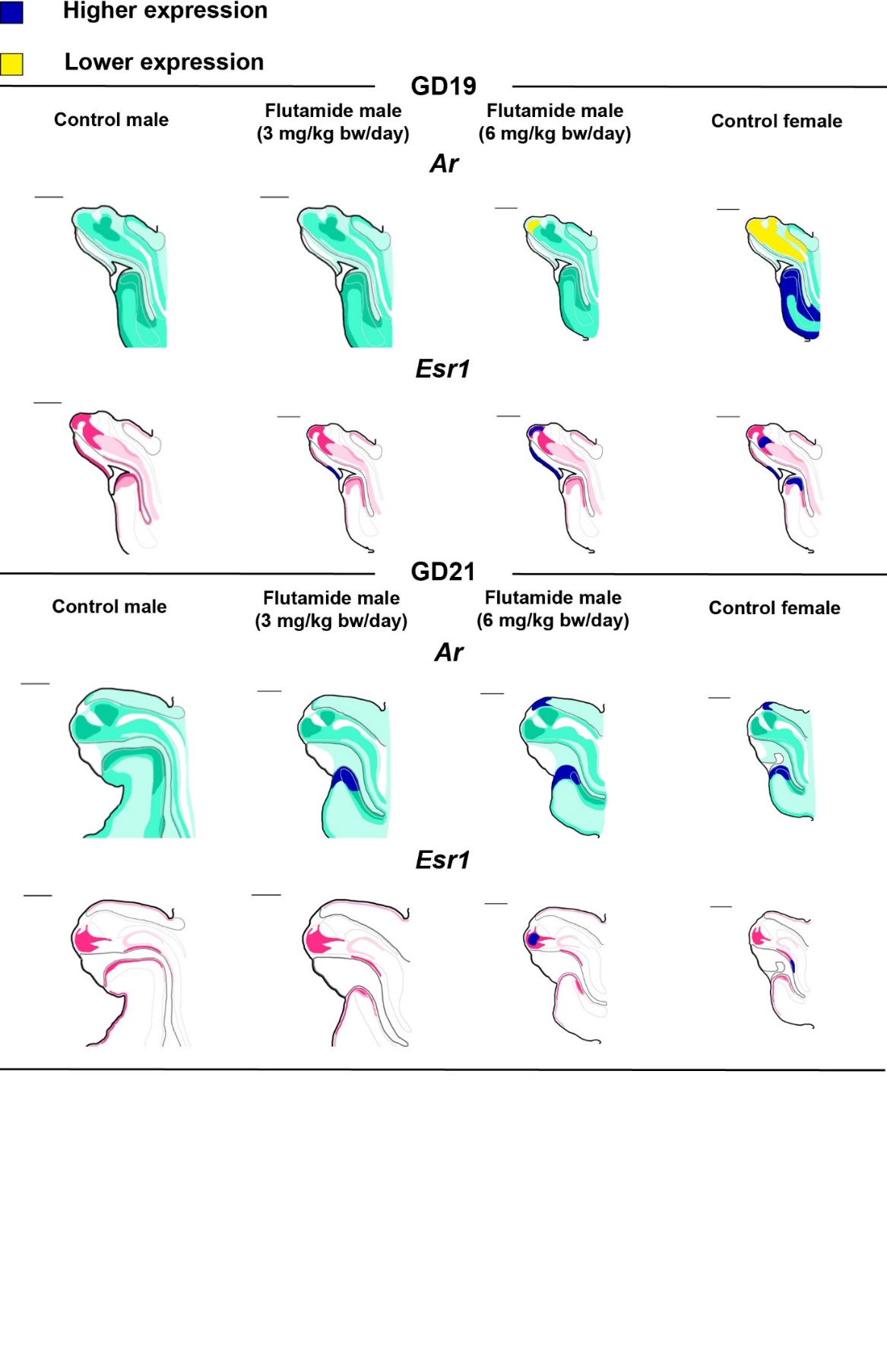


**Figure S1 Summary of *Ar* and *Esr1* expression changes at GD19 and GD21.** See figure 5 and 6 for the corresponding histological images. All changes are relative to control males (left column). Scale bar: 500 µm.


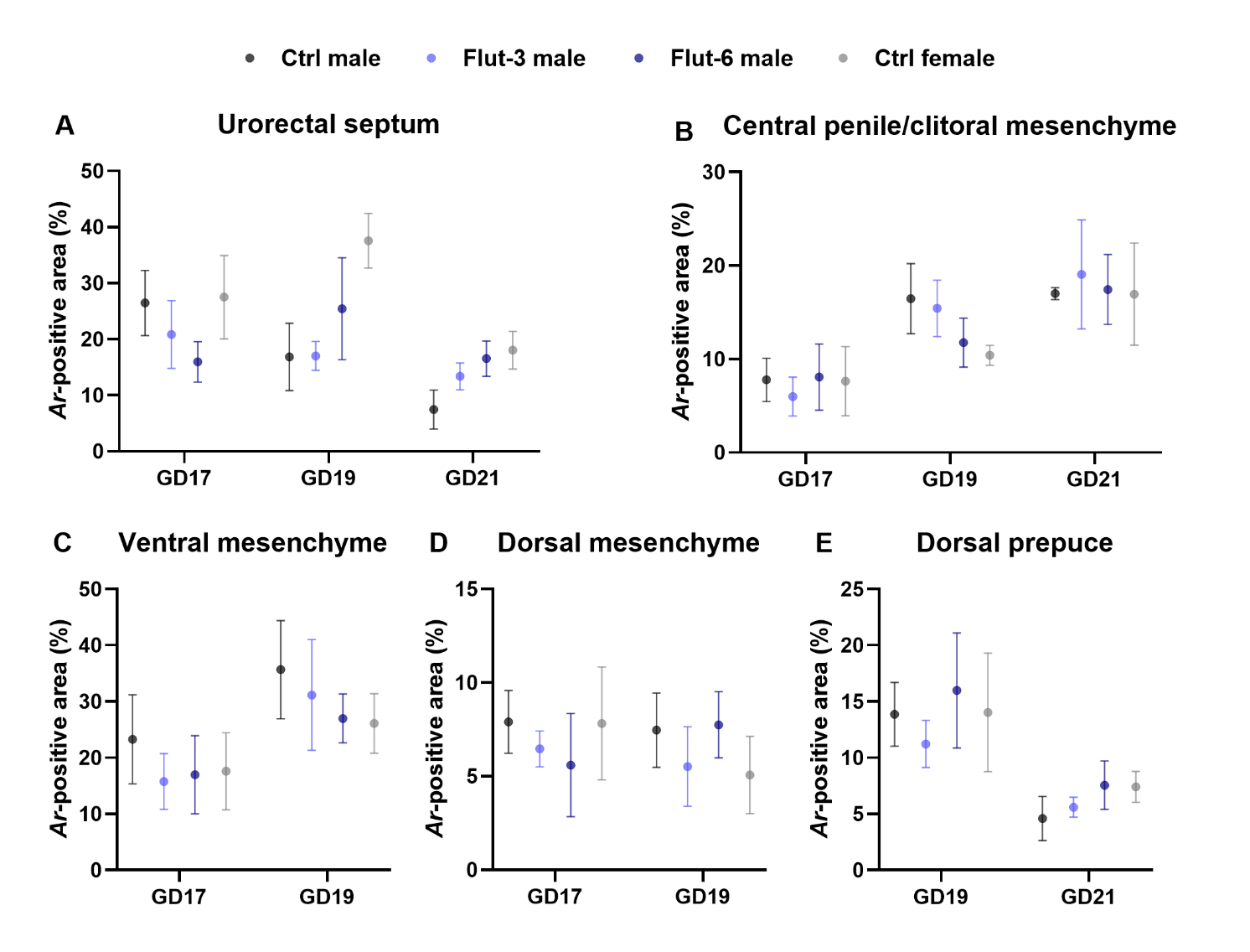


**Figure S2** **Semi-quantitative analysis of *Ar* expression in areas of the GT.** The percentage of *Ar*-positive area within a fixed region was semi-quantified in ImageJ. Data is shown as mean ± SD. No statistical analyses were performed.


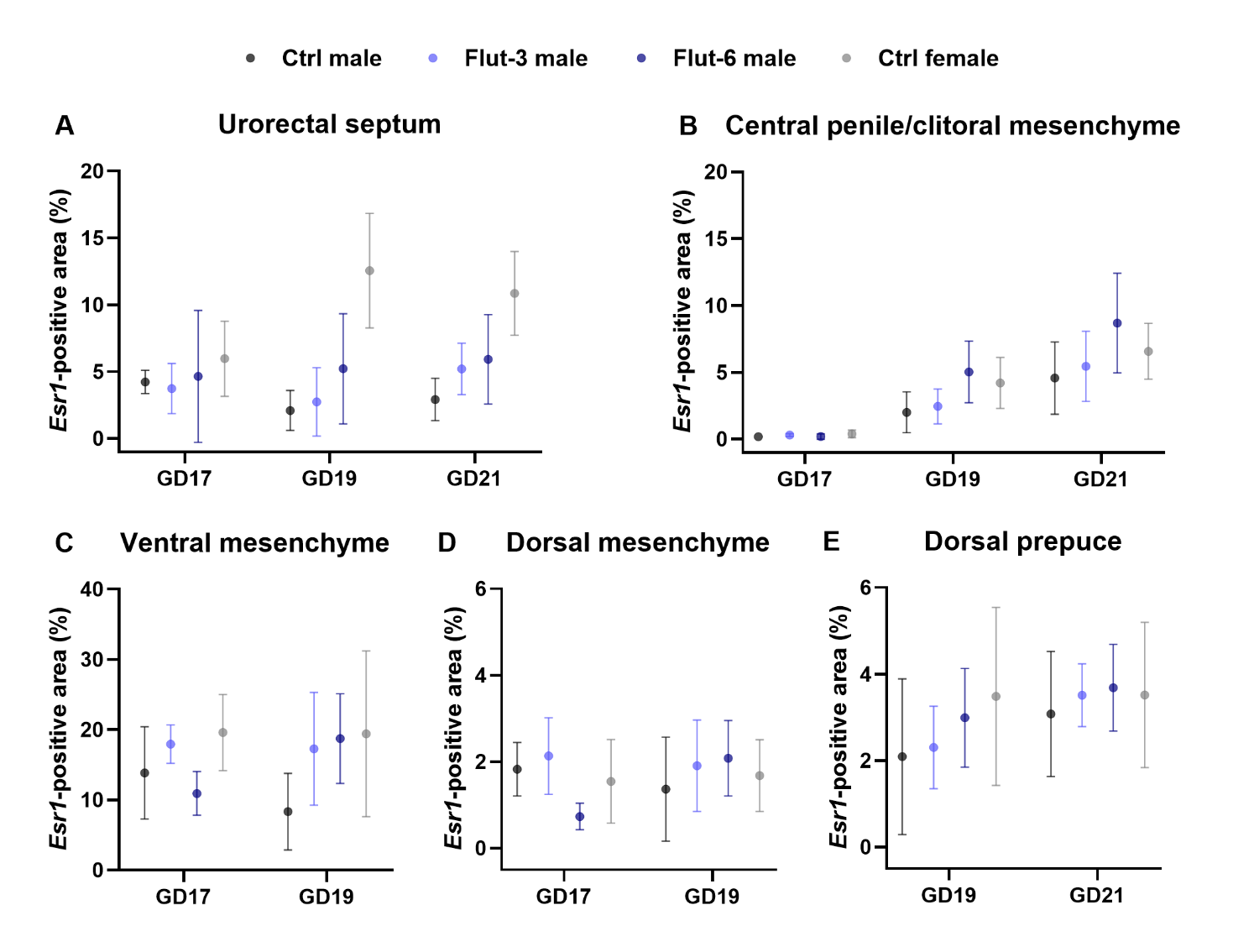


**Figure S3** **Semi-quantitative analysis of *Esr1* expression in areas of the GT.** The percentage of *Esr1*-positive area within a fixed region was semi-quantified in ImageJ. Data is shown as mean ± SD. No statistical analyses were performed.

## Supplementary Tables

**Table S1 Probes used for RNAscope® *in situ* hybridization and qPCR.**

| Probes for RNAscope Bio-Techne, Dublin, Ireland | | |
| --- | --- | --- |
| Target RNA | **Probe** | **Catalogue #** |
| *Esr1* | Rn-Esr1 | 317151 |
| *Ar* | Rn-Ar | 317141 |
| *Ppib* (positive control) | Rn-Ppib | 313921 |
| *DapB* (negative control) | DapB | 310043 |
| Gene expression assays for qPCR, Applied Biosystems®, Waltham, MA, USA | | |
| Target RNA | **Probe** | **Catalogue #** |
| *Esr1* | Rn01640372_m1 | 4331182 |
| *Ar* | Rn00560747_m1 | 4331182 |
| *Sdha* (housekeeping gene) | Rn00590475_m1 | 4331182 |
| *Rps18* (housekeeping gene) | Rn01428913_gH | 4331182 |

**Table S2 Dams and pregnancy data, postnatal rat toxicity study.** Dams were administered flutamide (6 or 18 mg/kg bw/day) or control vehicle by oral gavage from GD7-PD28. Data shown as mean ±SD. Statistical significance of postimplantation and perinatal loss were tested by Kruskal-Wallis test followed by Dunn’s post hoc test. For all other parameters, linear least square means regression and Dunnett’s post hoc test was used. **P<0.01.

^a^Postimplantation loss: Embryos lost prior to birth (implantations minus no. of pups)

^b^Perinatal loss: Embryos and pups lost before PD14 (Implantations minus no. of pups alive on PD14)

| Dams (postnatal animal study) | | | |
| --- | --- | --- | --- |
|  | **Control** | **Flutamide**  **(6 mg/kg bw/day)** | **Flutamide**  **(18 mg/kg bw/day)** |
| Number of litters | 12 | 11 | 11 |
| Body weight gain GD7 – GD21 (g) | 120.0 ±21.1 | 115.5 ±15.7 | 108.3 ±16.2 |
| Body weight gain GD7 – PD1 (g) | 31.4 ±11.2 | 27.7 ±24.6 | 24.6 ±13.3 |
| Litter size | 5.9 ±8.5 | 6.2 ±4.6 | 6.3 ±12.1 |
| Postimplantation loss (%)^a^ | 14.5 ±2.2 | 13.9 ±2.4 | 13.7 ±2.3 |
| Perinatal loss (%)^b^ | 11.4 ±10.3 | 11.6 ±6.8 | 6.8 ±11.9 |
| Body weight gain PD1-PD28 | -11.6 ±13.2 | -3.81 ±12.6 | -3.62 ±9.32 |
| Liver weight PD28 (g) | 11.3 ±1.4 | 11.8 ±0.9 | 12.1 ±0.9 |
| Liver weight PD28 (mg)/  body weight PD28 (g) | 39.3 ±3.4 | 41.0 ±2.1 | 42.6 ±2.1** |

**Table S3 Male offspring, postnatal rat toxicity study.** Dams were administered flutamide (6 or 18 mg/kg bw/day) or control vehicle by oral gavage from GD7-PD28. Data shown as mean ±SD. Statistical significance for nipples on PD14 was assessed using a generalized estimating equations (GEE) model. No statistical tests were used for hypospadias and testicular descend. For all other parameters, linear least square means regression followed by Dunnett’s post hoc test was used. For birth, PD6, PD14, and PD22 body weights, AGD, and AGDi, litter was included as a random and nested factor. Birth weight was additionally adjusted for number of pups in the litter. For organ weights, and PD16 and PD28 body weights, only one animal per litter was included in the analysis. In the 6 mg/kg/day flutamide dose group, 3 prostates, 5 seminal vesicles, and 1 levator ani muscle were below the detection limit, and the weight was imputed to 3 mg. Body weight was included as a co-variate for the organ weight analyses. **P<0.01, ***P<0.001.

| Male offspring (postnatal animal study) | | | |
| --- | --- | --- | --- |
|  | **Control** | **Flutamide**  **(6 mg/kg bw/day)** | **Flutamide**  **(18 mg/kg bw/day)** |
| Number of litters | 12 | 11 | 11 |
| PD1 | | | |
| Birth weight (g) | 7.2 ±0.6 | 7.1 ±0.8 | 7.0 ±0.6 |
| AGD (mm) | 3.6 ±0.3 | 2.4 ±0.3*** | 2.1 ±0.3*** |
| AGDi^a^ | 1.9 ±0.1 | 1.2 ±0.1*** | 1.1 ±0.1*** |
| PD6 | | | |
| Body weight (g) | 12.8 ±1.7 | 12.6 ±2.1 | 12.6 ±1.8 |
| Hypospadias | 0/12 | 11/11 | 11/11 |
| Penis weight (mg) | 12.8 ±1.3 | 8.5 ±1.5*** | 7.5 ±1.8*** |
| PD14 | | | |
| Body weight (g) | 28.2 ±4.0 | 27.1 ±5.3 | 27 ±4.8 |
| Nipples | 0.05±0.2 | 10.4±2.2*** | 12.0 ±0.2*** |
| PD16 | | | |
| Body weight (g) | 31.4 ±5.7 | 32.1 ±5.0 | 30.9 ±5.0 |
| Hypospadias | 0/21 | 21/21 | 22/22 |
| Paired testes weight (mg) | 98.0 ±12.5 | 104 ±17.8 | 79.5 ±10.5*** |
| Epididymis weight (mg) | 22.4 ±3.3 | 17.6 ±3.5** | 12.2 ±2.6*** |
| Penis weight (mg) | 35.6 ±3.6 | 20.8 ±3.5*** | 11.3 ±3.1*** |
| Prostate weight (mg) | 10.3 ±1.7 | 4.6 ±1.7*** | ^b^ |
| Seminal vesicles weight (mg) | 9.7 ±2.3 | 4.2 ±1.4*** | ^b^ |
| Levator ani muscle weight (mg) | 27.0 ±4.8 | 11.7 ±5.6*** | 2.8 ±1.8*** |
| PD22 | | | |
| Body weight (g) | 47.2 ±7.3 | 46.3 ±7.3 | 45.7 ±8.0 |
| Hypospadias | 0/21 | 18/18 | 19/19 |
| Penis weight (mg) | 45.6 ±4.1 | 26.8 ±5.1*** | 18.3 ±5.0*** |
| PD28 | | | |
| Body weight (g) | 75.5 ±12.9 | 73.9 ±12.2 | 76.2 ±12.0 |
| Penis weight (mg) | 57.4 ±9.7 | 33.3 ±6.1*** | 21.4 ±5.6*** |
| Testicular descend (PD14-PD28) | | | |
| Median day of testicular descend (if testes descended). | PD17 | PD18.5 | PD28 |
| Unilateral cryptorchidism | 0 | 14/33 | 14/22 |
| Bilateral cryptorchidism | 0 | 3/33 | 4/22 |

^a^AGDi=AGD/^3^√bw

^b^In the 18 mg/kg bw/day flutamide dose group, all prostates and seminal vesicles were either below the detection limit (7 prostates, 3 seminal vesicles) or completely missing from the animal (4 prostates, 8 seminal vesicles).

**Table S4 Female offspring, postnatal rat toxicity study.** Dams were administered flutamide (6 or 18 mg/kg bw/day) or control vehicle by oral gavage from GD7-PD28. Data shown as mean ±SD. Statistical significance was assessed by linear least square means regression followed by Dunnett’s post hoc test, including litter as a random and nested factor. Birth weight was additionally adjusted for number of pups in the litter. **P<0.01, ***P<0.001.

| Female offspring (postnatal animal study) | | | |
| --- | --- | --- | --- |
|  | **Control** | **Flutamide**  **(6 mg/kg bw/day)** | **Flutamide**  **(18 mg/kg bw/day)** |
| Number of litters | 12 | 11 | 11 |
| PD1 | | | |
| Birth weight (g) | 6.8 ±0.7 | 6.8 ±0.8 | 6.8 ±0.6 |
| AGD (mm) | 1.8 ±0.2 | 1.8 ±0.2 | 1.8 ±0.2 |
| AGDi^a^ | 1.0 ±0.1 | 1.0 ±0.1 | 1.0 ±0.1 |
| PD6 | | | |
| Body weight (g) | 12.2 ±1.8 | 12.3 ±1.8 | 12.0 ±1.8 |
| PD14 | | | |
| Body weight (g) | 27.7 ±2.3 | 26.7 ±4.6 | 26.4 ±3.8 |
| PD22 | | | |
| Body weight (g) | 46.3 ±7.5 | 46.3 ±6.9 | 43.9 ±7.1 |

^a^AGDi=AGD/^3^√bw

**Table S5 Dams and pregnancy data, prenatal rat toxicity study.** Dams were administered flutamide (3 or 6 mg/kg bw/day) or control vehicle by oral gavage from GD7 until necropsy at GD21. Data shown as mean ±SD. Statistical significance of postimplantation loss was tested by Kruskal-Wallis test followed by Dunn’s post hoc test. For all other parameters, linear least square means regression and Dunnett’s post hoc test was used. *P<0.05. **P<0.01.

| GD21 dams (prenatal animal study) | | | |
| --- | --- | --- | --- |
|  | **Control** | **Flutamide**  **(3 mg/kg bw/day)** | **Flutamide**  **(6 mg/kg bw/day)** |
| Number of litters | 7 | 7 | 8 |
| Body weight gain GD7 – GD21 (g) | 127.7 ±17.8 | 131.6 ±16.5 | 128.9 ±12.7 |
| Adjusted body weight GD21 (g)^a^ | 289.3 ±32.9 | 293.7 ±33.7 | 293.1 ±26.8 |
| Uterus weight (g) | 96.2 ±7 | 91.1 ±12.2 | 92.5 ±12.5 |
| Fetuses | 14.6 ±2.0 | 14.6 ±2.4 | 15.6 ±2.7 |
| Postimplantation loss (%)^b^ | 5.6 ±6.8 | 6.8 ±10.8 | 1.4 ±2.6 |
| Liver weight (g) | 11.6 ±1.6 | 13.2 ±1.4 | 13.6 ±1.3* |
| Liver weight (mg)/  adjusted body weight (g)^a^ | 40.7 ±1.9 | 44.9 ±2.1** | 46.5 ±1.7** |

^a^Adjusted body weight = Body weight - uterus weight

^b^Postimplantation loss: Embryos lost prior to birth (implantations minus no. of fetuses)

**Table S6 GD21 fetuses, prenatal rat toxicity study.** Dams were administered flutamide (3 or 6 mg/kg bw/day) or control vehicle by oral gavage from GD7 until necropsy at GD21.. Data shown as mean ±SD. Statistical significance was assessed by linear least square means regression followed by Dunnett’s post hoc test, including litter as a random and nested factor. Body weight was additionally adjusted for number of pups in the litter. ***P<0.001.

| GD21 fetuses (prenatal animal study) | | | |
| --- | --- | --- | --- |
|  | **Control** | **Flutamide**  **(3 mg/kg bw/day)** | **Flutamide**  **(6 mg/kg bw/day)** |
| Number of litters | 7 | 7 | 8 |
| Males | | | |
| Body weight (g) | 4.4 ±0.9 | 4.3 ±0.7 | 3.8 ±0.3 |
| AGD (mm) | 3.9 ±0.3 | 3.0 ±0.4*** | 2.6 ±0.3*** |
| AGDi^a^ | 2.4 ±0.2 | 1.9 ±0.2*** | 1.7 ±0.2*** |
| Females | | | |
| Body weight (g) | 4.2 ±0.7 | 4.0 ±0.6 | 3.6 ±0.3 |
| AGD (mm) | 1.9 ±0.2 | 2.0 ±0.0.2 | 2.0 ±0.2 |
| AGDi^a^ | 1.2 ±0.2 | 1.2 ±0.2 | 1.3 ±0.1 |

^a^AGDi=AGD/^3^√bw

**1.3 Supplementary videos**

Supplementary video 1: Control male GD21

Supplementary video 2: Control female GD21

Supplementary video 3: Flutamide (3 mg/kg bw/day) male GD21

Supplementary video 4: Flutamide (6 mg/kg bw/day) male GD21
